# Large-scale identification of human cerebrovascular proteins: Inter-tissue and intracerebral vascular protein diversity

**DOI:** 10.1371/journal.pone.0188540

**Published:** 2017-11-30

**Authors:** Soo Jung Lee, Soonhyung Kwon, John R. Gatti, Ejona Korcari, Ty E. Gresser, Princess C. Felix, Simon G. Keep, Kevin C. Pasquale, Tongxu Bai, Sabrina A. Blanchett-Anderson, Nancy W. Wu, Charissa Obeng-Nyarko, Kossi M. Senagbe, Kathy C. Young, Snehaa Maripudi, Bharath C. Yalavarthi, Dajana Korcari, Andre Y. Liu, Benjamin C. Schaffler, Richard F. Keep, Michael M. Wang

**Affiliations:** 1 Department of Neurology, University of Michigan, Ann Arbor, Michigan, United States of America; 2 School of Social Work, University of Michigan, Ann Arbor, Michigan, United States of America; 3 Department of Neurosurgery, University of Michigan, Ann Arbor, Michigan, United States of America; 4 Department of Molecular & Integrative Physiology, University of Michigan, Ann Arbor, Michigan, United States of America; 5 Neurology Service, VA Ann Arbor Healthcare System, Ann Arbor, Michigan, United States of America; Hungarian Academy of Sciences, HUNGARY

## Abstract

The human cerebrovascular system is responsible for regulating demand-dependent perfusion and maintaining the blood-brain barrier (BBB). In addition, defects in the human cerebrovasculature lead to stroke, intracerebral hemorrhage, vascular malformations, and vascular cognitive impairment. The objective of this study was to discover new proteins of the human cerebrovascular system using expression data from the Human Protein Atlas, a large-scale project which allows public access to immunohistochemical analysis of human tissues. We screened 20,158 proteins in the HPA and identified 346 expression patterns correlating to blood vessels in human brain. Independent experiments showed that 51/52 of these distributions could be experimentally replicated across different brain samples. Some proteins (40%) demonstrated endothelial cell (EC)-enriched expression, while others were expressed primarily in vascular smooth muscle cells (VSMC; 18%); 39% of these proteins were expressed in both cell types. Most brain EC markers were tissue oligospecific; that is, they were expressed in endothelia in an average of 4.8 out of 9 organs examined. Although most markers expressed in endothelial cells of the brain were present in all cerebral capillaries, a significant number (21%) were expressed only in a fraction of brain capillaries within each brain sample. Among proteins found in cerebral VSMC, virtually all were also expressed in peripheral VSMC and in non-vascular smooth muscle cells (SMC). Only one was potentially brain specific: VHL (Von Hippel-Lindau tumor suppressor). HRC (histidine rich calcium binding protein) and VHL were restricted to VSMC and not found in non-vascular tissues such as uterus or gut. In conclusion, we define a set of brain vascular proteins that could be relevant to understanding the unique physiology and pathophysiology of the human cerebrovasculature. This set of proteins defines inter-organ molecular differences in the vasculature and confirms the broad heterogeneity of vascular cells within the brain.

## Introduction

Technological advances have driven an increase in large-scale studies of molecular expression in the central nervous system. These studies provide important resources for the study of molecular networks that underlie brain function and neurological disease.

Available expression study data have been shaped by at least three factors. First, robust methods to quantify nucleic acids (most recently RNA-seq) have driven a marked expansion of transcriptomes in the nervous system. Proteomic analysis of the brain has lagged behind studies of gene expression since protein analysis is limited to lower throughput methods such as two-dimensional gel electrophoresis difference analysis combined with mass spectroscopy (individual studies reviewed by [1]). Second, cell types of focus in the brain are commonly neurons [2–5] and glia [6–9, 3, 10]; dedicated vascular expression studies [11, 12] compose a minority of brain datasets. Third, the popularity of rodent experimental models has driven the explosion of large scale expression studies of rat and mouse [11, 13, 12], with fewer studies focused on human brain. As a consequence of these three factors, it is not surprising that no studies, to our knowledge, include large scale profiling of proteins of the human brain vasculature.

Although vascular smooth muscle cells (VSMC) of the brain play a role in the genesis of human cerebrovascular disorders [14–16], no published studies to our knowledge examine the complement of proteins expressed in human brain VSMC. While there are ample studies showing differences between endothelial cells of the brain and of the peripheral organs [11, 12, 17], relatively little is known about cerebral smooth muscle cells. Thus, little is known about the molecular differences, if any, between human brain VSMC and peripheral vascular and non-vascular smooth muscle cells. Brain specific VSMC proteins might provide important therapeutic opportunities.

The Human Protein Atlas (www.proteinatlas.org) is an online resource of protein expression patterns that examines tissue expression of nearly all proteins in a panel of human tissues, including brain [18]. This database contains primary data from immunohistochemical analysis which enables any user to assess each protein for cellular expression patterns. Brain images from the Human Protein Atlas are sufficiently detailed to easily differentiate vascular cells from neurons and glia and to resolve expression between endothelial cells and VSMC.

In this study, we analyzed central nervous system protein expression patterns currently available in the Human Protein Atlas [18]. Specific objectives arising from this effort were: (a) to compare and contrast proteins expressed in human brain endothelial cells and VSMCs; (b) to discover the extent of vascular protein differences between the brain and other tissues (intra-organ molecular heterogeneity); (c) to characterize the extent of protein expression differences within vessels of the brain (intracerebral molecular heterogeneity); and (d) to assess the potential molecular differences between brain vascular and non-vascular smooth muscle cells in humans.

As a result of this effort, we have collated a new assembly of human proteins that are enriched in brain vascular cells over neurons and glia. We have performed validation studies of a subset of these markers, which support the reproducibility of methods used to generate brain data in the Human Protein Atlas. Most of these proteins have not previously been demonstrated in human brain vessels.

## Methods

### Protein expression scoring

Scoring of immunohistochemical images from the Human Protein Atlas was performed by a team of investigators. Subsets of human proteins were scored by a primary reviewer. Each vascular hit was then scored by two additional reviewers who were not involved in the original review of the data. Finally, all positives were reviewed by a final reviewer.

The workflow for the final review of vascular hits was performed as follows: 1) data available in December 2016 through January 2017 were examined; 2) clear vascular staining in the absence of strong and consistent non-vascular staining was assessed in cortex. Endothelial cell (EC) staining was scored when capillaries were stained without expression in perivascular cells; at the same time, in arteries, EC markers stained at least some arterial intima without non-intimal staining. Smooth muscle cell (SMC) staining was scored when there was absence of capillary endothelial signal and when the media of arteries was stained with clearly identifiable negative endothelial cells. Perivascular scoring included markers present outside the endothelium of capillaries and not present in astrocytes. Examples of novel brain vascular markers are shown in S1–S3 Figs. 3) assessments of cell distribution was made based on cortex staining (endothelial vs. smooth muscle), but due to the low prevalence of smooth muscle in brain, other brain regions were used if needed; 4) notation was made if all cortex samples had vascular staining; heterogeneity was assessed for capillary staining and was noted when more than between 15% and 85% of vessels showed strong staining; 5) notation was made if at least one sample from every brain region showed vascular staining; 6) to assess peripheral staining, we first examined smooth muscle sections, where an assessment of non-vascular SMC, vascular SMC, and EC staining was made. This was followed by examination of the gall bladder, colon, kidney, and lung. A cell type was scored as positive in these cell types if definitive evidence of staining above background was found in any of these tissues; 7) in cases where there was significant vascular SMC staining, it was sometimes difficult to ascertain EC staining; in these cases, by default, EC was scored positive if capillary staining was seen in brain; 8) perivascular staining in the brain was noted if the cells were not clearly capillary EC or SMC; these were mostly round cells outside the vessel (either pericytes or macrophages).

Tissue expression was then scored for all cerebrovascular hits by examining a series of organs that included the following: heart, lung, kidney, and liver. To assess for smooth muscle expression, gall bladder, colon, and esophagus were examined.

The final review of 490 initially flagged hits resulted in 346 final positives. Among those that did not make the final list, there were 110 proteins for which data was dropped from the HPA database and 32 proteins that on final review were not deemed convincing enough to score as vascular.

For assessments of EC tissue-to-tissue heterogeneity, we examined staining patterns for proteins that were present in EC of the brain but not in vascular SMC of smooth muscle tissues, gall bladder, colon, kidney, and lung. Proteins also expressed in SMC were difficult to score for presence or absence in EC when expression in both cells was similar.

### Validation analysis in human brain

We used selected antibodies employed by the Human Protein Atlas study that gave vascular expression patterns in brain for validation studies. We used conventional chromogenic staining on frontal cortex from at least 6 independent human brain samples. These were samples obtained at autopsy from patients with cerebral autosomal dominant arteriopathy with subcortical infarcts and leukoencephalopathy (CADASIL) and control brains from the University of Maryland Brain and Tissue Bank. Brain samples were fixed in formalin and then paraffin embedded prior to sectioning. The use of human brain samples obtained at autopsy has been reviewed by the VA Ann Arbor Heathcare System Research and Development Committee; the committee determined that this study was not considered human subject research regulated by its institutional regulatory board (IRB). The use of human brain samples was reviewed by the University of Michigan IRB which determined that this was research that is not regulated by the IRB. Consent from next of kin was obtained for all autopsies.

## Results

### Identification of proteins in brain EC and SMC in situ

The primary objective of this study was to comprehensively annotate proteins in the human brain that are expressed primarily in the vasculature of the human brain, relative to neurons and glia. The Human Protein Atlas offers an unprecedented collection of data demonstrating immunohistochemical localization of proteins in a wide collection of tissues. Four regions of the brain, the cortex, caudate, hippocampus, and cerebellum, are represented in this Atlas.

**Fig 1:**
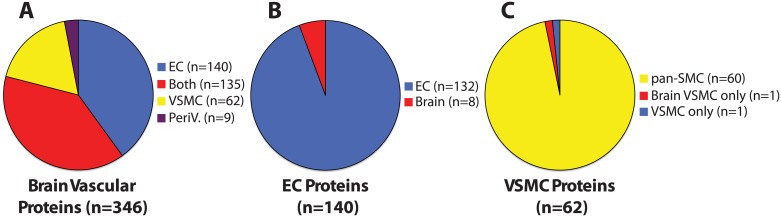
Summary of analysis of human brain vascular proteins curated from the Human Protein Atlas. To graphically demonstrate global findings, we present pie charts that summarize data shown in Tables 1–3 of this paper. (A) shows distributions of all proteins found primarily in vessels (Tables 1–4). (B) shows tissue distributions of endothelial cell (EC) proteins (Table 1) and vascular smooth muscle cell (VSMC) proteins (Table 3), respectively. (A) includes perivascular (PeriV) proteins listed in Table 4.

We examined all images available for brain expression, screening for proteins with vascular expression. From a total of over 20,000 queried proteins, we identified 346 proteins that formed a cerebral vascular-enriched protein set. Categorization of vascular proteins by cell type of expression demonstrated that most proteins were found in both EC and SMC of the brain (135), with some proteins showing only EC (140) or only VSMC (62) expression (Tables 1–3). Table 4 lists proteins that were expressed in perivascular cells but not in EC or VSMC of the brain. A summary of the results is shown in pie charts in Fig 1.

**Table 1:** Curated brain endothelial proteins from the Human Protein Atlas. Listed proteins (n = 140) were predicted to be expressed only in brain endothelium but not other cell types in the brain. A majority of these proteins are also expressed in non-cerebral smooth muscle.

A2M	CRYBA2	LBP	SCNM1
ABCC1	CXorf22	LMCD1	SDPR
ABCG2	CYP4F11	LMOD3	SLC16A1
AGFG1	DDX58	LONRF1	SLC22A15
AIF1L	DPF2	LRCH2	SLC2A1*
ALPL	EI24	LRRC8C	SLC30A1
ANKRD39	EIF4E1B	MAGEB5	SLC35B1
ANKRD58	ELOVL7	MFI2	SLC3A2*
ANPEP	EMP2	MRPS5	SLC6A12*
ANXA2	EPB41L2	MS4A10	SLFN12
APCS	EPS8L3	MS4A13	SMURF1
ARHGEF1	ESAM	MSN	TAPBP
ARHGEF5L*	ESPNL	MSRB3	TAS2R60
B2M	EXT1	MYADML2*	TCTEX1D1
BTD	FAIM3	NINJ1	TGM2
C10orf54	FAM100B	NLRP5	TJP1
C12orf49	FAM18B2	NME6	TMTC4
C13orf26	FBXL20	NRP2	TRAF6
CA4	FCN1	ORAI1	TRIM59
CABP7	GALR2	OSR1	TRIM73
CACNA1C	GBP4	PALMD	TRPV6
CAPN2	GGT5	PGLYRP4	TTLL5
CAPN8	GIMAP1	PHF21A	VAMP5
CCDC104	GIPC3	PI16	VAT1
CCDC48	GRRP1	PLA2G4F	WT1
CCDC71	HLA-E	PLXDC1	YES1
CD34	HSPA12B	PODXL	ZBTB5
CGNL1	HTR5A	PPP1R14D	ZFYVE28
CIB1	IFITM1	PRX	ZNF345
CLEC11A	IL17RC	PTH2R	ZNF557
CLEC14A	IL27	PTPN4	ZNF652
CLPS	INHBB	PTPRM	ZNF786
CNPY4	JAKMIP3*	PTTG1IP	
COLEC12*	KCTD19*	RABEP2	
COPG	KIAA2022	RLN3	
CRB3	LAP3	S100A10	

* proteins showing exclusive brain EC expression (and absent from EC from peripheral tissues).

**Table 2:** Proteins curated from the Human Protein Atlas that are expressed in both EC and VSMC of the brain. All proteins (n = 135) are predicted to be expressed in both types of the two principle types of vascular cells but not other cells of the central nervous system.

AAMP	CITED4	IQGAP1	PID1	TIAF1
AEBP1	COBLL1	IQSEC1	PNRC2	TINAGL1
AGAP1	CRIP2	ITPRIPL1	PPEF2	TMEM164
ALG1L	CTDSPL	JMJD4	PRKRIP1	TNS1
ALG5	CUEDC2	KANK3	PTPRK	TPCN2
ANKRD20A2	CYB561	KBTBD6	PTRF	TRIM52
ANUBL1	CYB5D1	KLHL38	PVRL2	TSC1
ARHGAP18	DAG1	KIAA1430	QTRT1	UBTD1
ARHGAP20	DCHS2	LAMA2	RAB32	UTRN
ATP6V0A2	DGKG	LAMB2	RAPH1	WBSCR27
AVPI1	DHRS7C	LDLRAD2	RBM20	ZCCHC11
BCAM	DOCK1	LIMS2	RELA	ZDHHC6
BFSP1	EGFL8	LMTK3	RELL1	ZNF7
BMPR1A	EHBP1L1	MAGEB10	RNF39	ZNF827
BNIP2	EHD2	MAS1L	RP2	ZZZ3
BPNT1	EHD3	MGLL	Rsu1	
C10orf71	ELMOD3	NAB1	RTN4RL1	
C11orf60	EVI5L	NANOS1	S100A6	
C19orf54	FAM166B	NES	SERPINH1	
C19orf60	FAM175B	NMB	SGCB	
C1orf163	FLNA	NSMCE1	SHC1	
C1orf175	FUZ	NUS1	SLC30A7	
C9orf47	GALNT10	NXPH1	SLC45A4	
C9orf71	GPER	OR11H1	SLC6A9	
C9orf75	GRAMD1C	OR5A2	SNAP47	
CCDC140	GTSE1	OR6B2	SNX1	
CCNYL1	HIP1	PAK4	SPINT1	
CD151	IFITM2	PAWR	SYNPO2	
CD59	IFITM3	PDGFA	SYNPO2L	
CFH	ILK	PICALM	TEKT5	

**Table 3:** Brain vascular smooth muscle proteins curated from the Human Protein Atlas. Proteins (n = 62) predicted to be expressed only in vascular smooth muscle of the brain but not in EC of the brain are listed. All proteins stains localized to vascular cells but not substantially or consistently in other cells of the central nervous system. Unless indicated, all proteins were found in both non-brain VSMC and non-vascular SMC.

ADCY5	CT47A11	LITAF	S100A4	ZNF527
AGXT	DDX59	LPHN2	SH3BGRL3	ZNF704
AHSA2	DES	LRRC41	SPANXA1	
ALDH3B2	DMRTC1B	MAP7D3	SPANXB2	
AOC3	ENOX2	MTFR1	ST6GALNAC6	
AOF1	EPX	NBEAL2	SUSD5	
ARGFX	FAM124A	NEURL4	TBC1D2B	
ASPG	FAM161A	NIPBL	TEX261	
C10orf116	FBXW4	PARG	TSPYL1	
C10orf4	GAB3	PBRM1	TXLNB	
C17orf78	GPX8	PLA2G15	UNK	
C9orf152	GZMM	PRMT2	VHL*	
CCDC82	HRC**	PTMS	YIF1A	
CHRNA5	IFNA2	RAPSN	ZC3HAV1L	
CLEC19A	KIAA1984	RHOF	ZNF197	

* protein only found in brain vascular smooth muscle (not in other VSMC or in non-vascular SMC).

** proteins found in VSMC but not in other SMC.

**Table 4:** Brain perivascular proteins curated from the Human Protein Atlas. Proteins to be expressed in brain perivascular cells but excluded from EC and SMC are listed (n = 9). Proteins in perivascular compartments but also in other vascular cell types are listed in Tables 1–3.

ABCA8	INPP5K
C1QTNF1	MRPL32
CBY3	SLC19A1
CTSH	ULBP2
IFI30	

### Validation studies

Among the 346 vessel-positive proteins, we found that 94.6% of antibody stains (n = 31; one antibody did not stain at all) were concordantly positive between multiple cortex samples. Also, in general, positive staining of the cortex was predictive (95.7% for hippocampus, caudate, and cerebellum) of staining in other regions of the brain within the database. Very few proteins showed differential expression between brain regions, but some (e.g. COX16 that was not in cortex) were regionally distributed. These observations suggested that in the conditions used to generate brain images for Human Protein Atlas, there was high reliability.

To test the generalizability of the Human Protein Atlas findings, we used a subset of the same antibodies to assess independent human brain samples obtained at autopsy. Our results were remarkably concordant with those of the Human Protein Atlas, reaching 98.07%(51/52) agreement for vascular staining. We conclude that the Human Protein Atlas brain staining results are highly predictive of patterns of brain staining in an independent lab. Examples of validation stains are shown in S1–S3 Figs.

### Tissue specific expression of vascular proteins within the brain

The brain circulation demonstrates unique properties, including strong barrier function that results in the blood brain barrier (BBB) and activity dependent flow regulation. Since it is possible that these brain vascular physiological functions could be mediated by proteins unique to the brain vasculature, we searched for protein expression patterns found only in brain vascular cells. Comparison of brain to peripheral tissues revealed that vascular expression of 9 proteins was unique to the central nervous system. A majority of these proteins (8/9) were found in EC only. These proteins are highlighted in Table 1.

### Caliber specific EC proteins of the brain

Since human brain sections contained vessels of a variety of calibers, we also scored for endothelial proteins that were differentially expressed by vessel size (Table 5). A small fraction of EC proteins were found in capillaries but were not found in larger vessels, including the small arteries of the brain. Another small group of EC proteins was found in a complementary pattern; these proteins were expressed in the intima of small arteries but not found in capillaries.

**Table 5:** Proteins expressed in endothelial cells of different sized vessels. Proteins in EC or EC/VSMC that displayed differences in expression according to the size of the vessel are shown.

ANPEP*	MSRB3**
CCDC71*	PRX*
CCNYL1**	S100A6**
EHBP1L1**	TEKT5**
GPER**	ZBTB5*
KCTD19*	

* proteins that are expressed in capillaries of the brain but reduced in expression in EC of arteries.

** proteins expressed in arterial but not capillary EC of the brain.

### Expression of SMC markers in brain VSMC, non-brain VSMC, and in non-vascular SMC

Data from the Human Protein Atlas were used to investigate expression overlap between brain and peripheral VSMC expression markers. We also queried overlap between brain VSMC and non-vascular SMC proteins. After identification of proteins expressed in brain VSMC (but not brain EC; Table 3), we compared the distribution of these proteins in peripheral human tissues, including smooth muscle predominant organs such as the uterus and bladder, gall bladder, and colon, with special attention to VSMC expression and to non-vascular SMC. The overwhelming majority of brain VSMC expressed markers were found in both vascular SMC and in non-vascular smooth muscle; these proteins thus define the smooth muscle phenotype (60 proteins; so-called pan-SMC proteins) and do not distinguish between VSMC and non-vascular SMC. Only VHL (Von Hippel-Lindau tumor suppressor) was found in brain VSMC but not non-brain VSMC. Among the brain VSMC proteins, only two proteins were expressed in VSMC but not non-vascular SMC (HRC [histidine rich calcium binding protein], and VHL). With a single exception (PCDHB11 [protocadherin β-11]), proteins identified in VSMC of peripheral organs were also found in brain VSMC. Thus, the vast majority of VSMC proteins identified are present both in brain and non-brain VSMC.

We next analyzed whether markers found in both EC or VSMC of the brain could discriminate between VSMC and non-vascular SMC of peripheral organs. Surprisingly, eight proteins of brain EC/VSMC proteins were absent from non-vascular SMC (Table 6; top). Finally, we analyzed brain EC proteins for expression in VSMC and non-vascular SMC. Fifteen EC proteins were present in at least one non-vascular SMC but were absent from VSMC (Table 6; bottom).

**Table 6:** Proteins that show differences between VSMC and non-vascular SMC.

ABCG2**	MS4A13**
AGAP1*	PALMD**
ALG1L*	PID1*
BCAM*	PTPN4**
CABP7*	SDPR**
DDX58**	SLC16A1**
EIF4E1B**	SLC22A15**
ELOVL7**	SLC30A1**
FAM18B2**	TMTC4**
ITPRIPL1*	YES1**
KIAA2022**	ZZZ3*
MS4A10*	

* proteins found in brain or non-brain VSMC that are absent from non-vascular SMC.

** proteins from Table 1 (found in brain EC) that are present in at least one non-vascular SMC (from gastrointestinal tissue or endometrium) but absent from VSMC (in all tissues including brain).

To summarize, only two brain VSMC proteins were unique to VSMC. On the other hand, 23 brain EC/VSMC proteins were differentially expressed between VSMC and non-vascular SMC.

### Intracerebral heterogeneity of vascular markers

**Fig 2:**
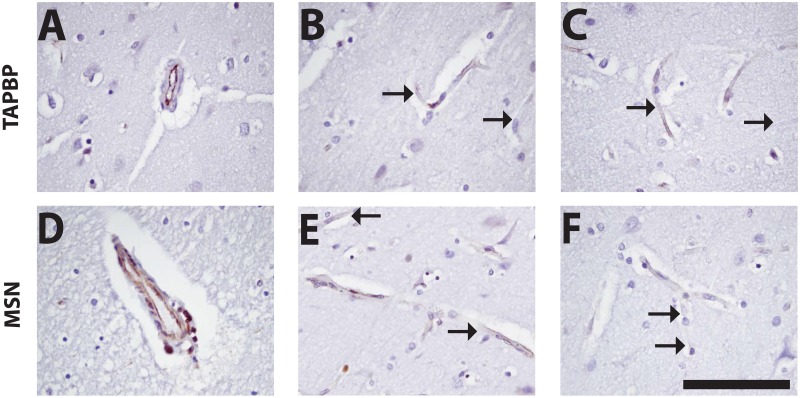
Heterogeneity of specific protein markers in human brain vessels. Human cortex samples were stained for TAPBP(tapasin) and MSN (moesin) to confirm intracerebral heterogeneity of marker expression. These markers showed staining in less than 85% of vessels in both Human Protein Atlas images and in validation studies performed by us. In both of these cases, EC staining for each protein varied from vessel to vessel, particularly in capillaries. A and D show relatively uniform expression in EC, with MSN missing only a small number of cells. In B, C, E and F, frequent unstained EC were found that are highlighted with arrows. Scale bar represents 100 um and is applicable to all panels.

Recent work has shed light on heterogeneity of EC cell types [19–23]. To assess the extent of cell-to-cell heterogeneity of the markers identified from this study, we scored whether markers were pan-EC (>85% capillary coverage) vs. heterogeneously expressed in human brain cortex images from the Human Protein Atlas (Table 7). Fig 2 shows examples of heterogeneous expression patterns for several of the markers; in capillaries, some of these markers exhibit marked differences in adjacent cells. In general, the heterogeneity of protein expression in capillaries was even more marked in caudate and hippocampus than in cortex.

**Table 7:** Brain EC proteins displaying intracerebral heterogeneous expression. Proteins (n = 16) were identified as heterogeneously expressed if in all cortex samples examined, capillary EC displayed highly variable levels of staining.

ALPL	CRB3	GIPC3	PTPRM
BPNT1	CYB561	MSN	SLC35B1
CA4	EHD3	PRKRIP1	SLC45A4
CABP7	FCN1	PTH2R	TAPBP

### Inter-organ heterogeneity of vascular markers

**Fig 3:**
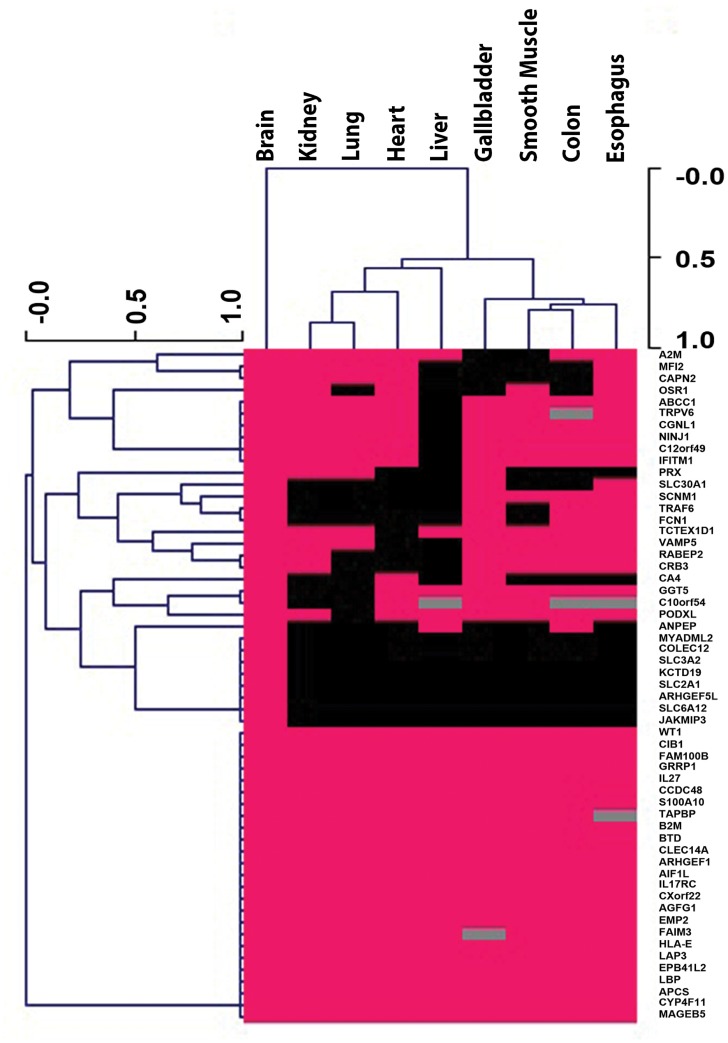
Cluster analysis of brain endothelial proteins identified by Human Protein Atlas analysis. Proteins with expression in cerebral endothelial cells (without smooth muscle expression in any tissue; a subset selected from Table 1) were scored for non-cerebral EC expression in tissues shown. Expression in these tissues was given a binary score (yes [pink] or no [black]) and cluster analysis was performed to identify patterns of tissue expression using the MeV available online (mev.tm4.org) to display hierarchical clustering of tissue distributions (on the x-axis) and clusters of proteins in similar distributions (on the y-axis). Some expression data was not available from the Human Protein Atlas (grey boxes).

Brain vessels have unique properties, but it is expected that they share a large degree of molecular similarity with peripheral vessels [11]. To assess the similarities of marker expression between the brain and other major organs, we determined the expression of 59 brain EC proteins listed in Table 1 in kidney, lung, heart, liver, gall bladder, uterus, colon, and esophagus. (We excluded from this analysis proteins expressed in peripheral VSMC since it was difficult to differentiate VSMC and EC expression with certainty in all cases.) As noted above, a minority of these proteins were only expressed in brain EC, though brain only EC proteins were the largest organ pattern observed. The remainder of the markers was clearly expressed in 4.8 (on average) of 9 tissues examined. A largest category of organ involvement that included more than just brain was pan-expression in all endothelial beds. Fig 3 shows that cluster of markers are oligospecific for endothelial cells in broad categories. A large number of tissue groupings were represented, but some patterns were not common (eg. oligospecific patterns involving the brain and the liver were much less common than for other organs). These finding demonstrate that most brain EC markers are shared in complex patterns with other tissues.

## Discussion

The availability of large databases of protein expression is a promising advance. We show that large-scale analysis of one such dataset, the Human Protein Atlas, can be used to identify a cohort of new human brain vascular proteins. Analysis of the substantial assembly of proteins described herewith supports several interpretations: 1) brain-specific vascular proteins overwhelmingly localize to EC rather than SMC; 2) brain EC proteins exhibit wide degrees of interorgan and intracerebral heterogeneity; and 3) virtually all brain SMC markers are shared with peripheral VSMC and non-vascular SMC.

### Advantages of approach

Our approach leverages a mature, comprehensive set of data available online to curate assemblies of proteins expressed in the human cerebrovasculature. The design of the study offered several advantages over previous work that has described large scale molecular characterization of the brain vasculature.

This study provides large scale information about protein expression which complements an abundant amount of transcriptome data (Allen Brain and RNAseq data). It is clear that RNA expression may not always correlate with the presence of the gene product in cells. In addition, analysis of this data is cost efficient and easy to perform and does not require specialized equipment or samples, since images are publically available. As such, this approach is generalizable to other organ systems and other cell types within specific tissues. Notably, human tissue analysis sidesteps potential limitations of using model organisms, which can express different sets of proteins compared to humans [24].

A technical advantage of our approach is that it does not require cell separation methods, because we visually scored protein expression in minority cell populations in situ within a complex tissue such as the brain; in contrast, in prior work, vessel tissue from experimental animals was purified after tissue disruption, enzymatic treatments, or cell sorting procedures; it is unclear if the cells from animals undergo changes when isolated in this fashion.

In this study of vessels, analysis of human brain offers a further advantage in that cell types that compose the vasculature are larger and, hence, easier to differentiate compared to those in rodent vessels. In rodent brain [12], most vessels are capillaries with a dominance of endothelial cells, and there is a dearth of perivascular and smooth muscle cells. In contrast, human brains contain a diverse family of vessel sizes and types. The larger arteries of the leptomeninges contain easy to distinguish layers. The ease of interpretation allowed us to efficiently categorize vascular brain proteins by cell type of expression in a single view.

We also were able to compare vascular cell expression in multiple tissues using the Human Protein Atlas. The availability of over 30 tissues, stained with the same antibodies as used in brain analysis, offers a very efficient ability to compare the expression pattern in different parts of the body. So far, comparative transcriptome analysis of rodents has been limited to small numbers of tissues.

Finally, we emphasize that our approach is based on open access data; the information is available to all investigators world-wide and updates are easily viewed using a web browser at no cost. This makes it possible for users to verify our data and update findings using additional images that are uploaded on a continuous basis. Ongoing, crowd-sourced verification (analogous to Wikipedia editing) of our analysis should, in theory, make it possible to correct individual mistakes in our analysis that are unavoidable in large dataset analyses.

### Limits of approach

While the current Human Protein Atlas contains cell expression annotations, we found these were not satisfactory for identifying vascular enriched proteins in the brain. Therefore, for our screen, we manually inspected imaging data. The sheer volume of data analyzed amounted to millions of images and presented a significant challenge. We overcame this difficulty by utilizing a large team of investigators to manually curate the database. For some laboratories with specific questions, automated image pattern recognition with machine learning could be considered.

A recurrent concern in large scale analysis is the potential for error and challenges with reproducibility of data. Our experiments assessed the reproducibility of the immunostains for a selected group of probes used in the Human Protein Atlas. We found that we were able to reproduce immunostaining patterns with remarkable success. In addition to replicating cell expression patterns, we showed a consistent pattern of expression in brains that had been obtained under different conditions. These finding support high confidence in the utility of the Human Protein Atlas dataset.

But, perhaps the most significant potential disadvantage of this approach is that protein localization is strictly dependent on the quality of antibodies used for the study. Antibodies do not offer bias free detection as do nucleic acids. Clearly, heterogeneity of antibody reagents limits the scope of our protein screen. Future work on specific proteins identified in this study will require verification of expression using additional independent probes and/or validation of localization using in situ hybridization.

### Brain EC heterogeneity

EC of the brain have recently been shown to exhibit heterogeneous expression of molecular markers [19–23]. The first indications of this were discovered in genetically modified mice in which transgene expression was found to have different levels of expression in cerebral capillaries [19]. Transcripts for DLL4 in retinal vessels are also heterogeneously expressed [25]. In humans, the ABO blood group antigens exhibit diverse cell expression [22]. This study highlights a largely expanded set of markers, which are expressed in heterogeneous patterns in the same brain sections. This molecular heterogeneity seems to be a widespread property generalized to many markers, including the new molecules identified in this study. The pool of heterogeneously expressed proteins suggests that coordinate patterns of expression could define several different EC types within the brain. An alternative explanation is that cells exhibit distinct molecular patterns due to environmental cues. Molecular heterogeneity of brain EC could present some challenges and opportunities for translational research: targeting one specific EC cell type may not be sufficient as an approach to disease treatment or prevention; on the other hand, markers may define target populations for alteration of endothelial cells in specific conditions.

### Oligospecific EC expression patterns

To determine whether EC proteins from the brain were more similar to sets of peripheral EC, we analyzed a subset of EC proteins from Table 1. We focused on 59 EC proteins that were only expressed in EC of the vasculature (ie. absent from both brain and peripheral VSMC). These EC proteins were expressed in multiple patterns across nine different tissues, revealing wide heterogeneity between organs. Because of the specialized properties of cerebral EC, great efforts have been devoted to finding BBB specific markers.

Our studies did identify several EC markers that were specific to the brain, but most EC markers were also expressed in several peripheral organs in EC. A clear EC pattern that defines the nearest related peripheral EC did not emerge, but there were multiple complex and overlapping tissue expression patterns. It is possible that tissue specific EC phenotypes could be determined by combinations of proteins that are not EC restricted or were not found in this analysis. Of the eight brain EC-specific proteins, three were members of the solute carrier (SLC) family of transporters, SLC2A1, SLC3A2 and SLC6A12. SLC2A1, GLUT1, is a facilitative glucose transporter highly expressed at the BBB that supplies the glucose requirements of the brain [26]. SLC3A2, 4F2 heavy chain, is a component of many amino acid transporters including LAT1, a large neutral amino acid transporter present at the BBB that supplies the brain with essential amino acids [27]. SLC6A12, BGT-1, is a betaine/γ-aminobutyric acid (GABA) transporter that has been identified at the mouse BBB [28]. Apart from transporting important physiological substrates, it may also be important in regulating cell volume. However, apart from these transporters, five others were identified as being brain EC-specific proteins. These were ARHGEF5L, COLEC12, JAKMIP3, KCTD19 and MYADML2. The role of these proteins at the BBB is unknown and merits investigation. It is interesting, however, that mutations in COLEC12, collectin subfamily member 12 (CLP1), have been associated with profound alterations in brain development [29] and diabetic retinopathy [30].

Online Human Protein Atlas data also confirmed human cerebral EC expression of PECAM (CD31) and ABCB1 (p-glycoprotein/MDR). As canonical EC markers, these two proteins were not included in Tables, which were intended to highlight novel markers. Staining for CD31 was found in non-brain EC, while ABCB1 was found primarily in cerebral EC. It is interesting to note that PECAM staining in human brain met criteria for a heterogeneous EC protein.

### VSMC proteins in the brain

Few large scale molecular investigations have incorporated cerebral SMC, which makes up a small component of the cerebral vasculature in mouse. Although human SMC are also a minority of the vascular cell population in humans, most sections of brain tissue in the Human Protein Atlas contain sufficient numbers of cells, enabling the discovery of a new SMC protein marker set.

Among proteins found in cerebral SMC, we found that virtually all were also expressed in non-vascular SMC, including bladder and uterus. This agrees well with previous studies that show that among proteins described as vSMC markers, the vast majority are found in non-vascular SMC as well [31–35]. Collectively, these data support the remarkable similarity between vascular and non-vascular tissue. This contrasts markedly with the newly described EC markers, which demonstrated much higher inter-organ variability.

Since vascular smooth muscle is expected to have altered functions compared to non-vascular smooth muscle, there should be proteins that distinguish the two SMC lineages. For example, vimentin is expressed in vascular but not non-vascular SMC [36]. Our analysis identified several new proteins that are differentially expressed between vascular SMC and non-vascular SMC (Table 6). These markers may be useful resources for investigations on development of the vascular SMC phenotype. Interestingly, all of these proteins (like vimentin) are also expressed in EC, and there was only a single SMC-only (not EC) marker, HRC, found that differentiated between vascular and non-vascular SMC. HRC encodes a sarcoplasmic reticulum protein that was previously found in skeletal and cardiac muscle [37]. Going forward, this cerebral SMC protein data is important because several small vessel disorders (eg. CADASIL) are caused by degeneration of SMC [38, 39]; these cerebral small vessel diseases, as a rule, do not affect tissues that have large components of non-vascular SMC. Further experimental studies will be needed to determine if any VSMC markers are differentially expressed in different vascular beds, as has been reported for DES [40].

### Protein families and potential signaling pathways in brain vascular cells

Several categories were represented by multiple structurally related proteins among brain vascular markers identified in our screen (Table 8). Proteins of the SLC family have been described extensively in vessels and represent the largest category of proteins we found in vascular cells. These proteins have been described in endothelial cells of the brain where they may participate in specialized blood brain barrier functions [41–43]. The recurrent identification of multiple members of these groups is therefore consistent with prior work. In addition, we identified a series of zinc finger proteins, which could participate in transcriptional regulation. The brain EC expression of these factors suggests a potential master role in controlling expression of other brain vascular factors. Surprisingly, other families of proteins were commonly found, including olfactory receptors; a previous report has described OR10J5 in arterial and venous EC [44], where it apparently participates in cell signaling and migration. Additionally, proteins previously thought to be enriched or specific to the testis were strongly represented among brain vascular markers. Finally, a notable fraction of proteins found in brain vessels, but not in other brain components, were initially described as components of the inflammatory system.

**Table 8:** Structurally related grouping of brain vascular markers identified from the Human Protein Atlas. Clusters of structurally related proteins were identified in brain vessels and are listed in groups that include the SLC, TRIM, CCDC, ZNF, and OR protein families.

	Protein	Description
**SLC Family Protein**	SLC16A1	Monocarboxylate transporter 1
	SLC19A1	Folate transporter 1
	SLC22A15	Solute carrier family 22 member 15
	SLC2A1	Solute carrier family 2, facilitated glucose transporter member 1
	SLC30A1	Zinc transporter 1
	SLC30A7	Zinc transporter 7
	SLC35B1	Solute carrier family 35 member B1
	SLC3A2	4F2 cell-surface antigen heavy chain
	SLC45A4	Solute carrier family 45 member 4
	SLC6A12	Sodium- and chloride-dependent betaine transporter
	SLC6A9	Sodium- and chloride-dependent glycine transporter 1
**TRIM Family Protein**	TRIM52	Tripartite motif-containing protein 52
	TRIM59	Tripartite motif-containing protein 59
	TRIM73	Tripartite motif-containing protein 73
**CCDC Family Protein**	CCDC104	Cilia- and flagella-associated protein 36
	CCDC140	Coiled-coil domain-containing protein 140
	CCDC48	EF-hand and coiled-coil domain-containing protein 1
	CCDC71	Coiled-coil domain-containing protein 71
	CCDC82	Coiled-coil domain-containing protein 82
**ZNF Family Protein**	ZNF197	Zinc finger protein 197
	ZNF345	Zinc finger protein 345
	ZNF527	Zinc finger protein 527
	ZNF557	Zinc finger protein 557
	ZNF652	Zinc finger protein 652
	ZNF7	Zinc finger protein 7
	ZNF704	Zinc finger protein 704
	ZNF786	Zinc finger protein 786
	ZNF827	Zinc finger protein 827
**Olfactory Family Protein**	OR11H1	Olfactory receptor, family 11, subfamily H, member 1
	OR5A2	Olfactory receptor, family 5, subfamily A, member 2
	OR6B2	Olfactory receptor, family 6, subfamily B, member 2

We also performed functional group analyses of vascular proteins identified in the Human Protein Atlas. The top pathways suggested by PANTHER analysis are shown in Table 9, along with protein member that participate in each process. While some molecular pathways were expected (angiogenesis, integrin signaling), other pathways suggested by this analysis have not been previously identified in vascular cells (CCK, GnRH signaling).

**Table 9:** Functionally-related groups of brain vascular markers identified from the Human Protein Atlas. Clusters of functionally related proteins were identified in brain vessels using PANTHER analysis and are listed in groups that contain more than 3 proteins. Proteins related to calcium regulation are also included.

Pathway	Protein Name
**Inflammation Mediated by Chemokine and Cytokine Signaling Pathway**	ADCY5
	PAK4
	RELA
	SHC1
**Integrin Signaling**	DOCK1
	FLNA
	ILK
	LAMA2
	LAMB2
	LIMS2
	SHC1
**Gonadotropin-Releasing Hormone receptor**	BMPR1A
	CACNA1C
	INHBB
	NAB1
	RELA
	SLC2A1
**CCKR Signaling**	SHC1
	TPCN2
	TRAF6
	YES1
**Huntington Disease**	CAPN2
	CAPN8
	HIP1
	VAT1
**Nicotine Pharmacodynamics**	CACNA1C
	CHNA5
	EPB41L2
	FLNA
**TGF-beta Signaling**	BMPR1A
	CITED4
	INHBB
	SMURF1
**Angiogenesis**	HSPB2
	PDGFA
	SHC1
**Proteins related to calcium regulation**	CABP7
	CACNA1C
	CIB1
	HRC
	ORAI1
	TPCN2

**Fig 4:**
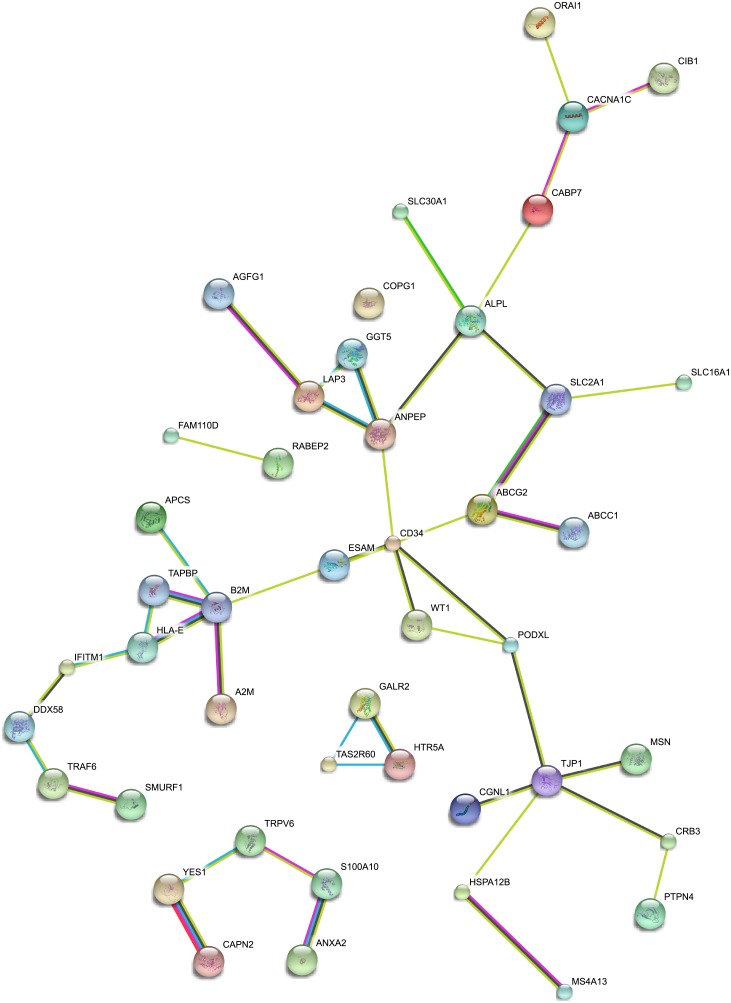
Protein interaction networks among vascular proteins identified from the Human Protein Atlas. We applied the STRING tool (string-db.org; Version 10.0) to human brain EC proteins to identify possible novel networks among molecules. The graphical representation of proteins identified as having at least one connected partner in the EC protein set are presented here. Connections included both physical interactions, co-expression, and text-mined connected proteins, and only interactions that were consider medium to high confidence were included. The same analysis, applied to SMC proteins, yielded only limited protein interactions.

Interaction networks were queried among EC and SMC proteins (Fig 4) and revealed modest interactions between EC proteins. Of note, nodes of interactions were found around CD34, YES1, TJP1, and B2M. Only one of the brain endothelial enriched proteins, SLC2A1 (GLUT1) has been described as an interactor with other endothelial molecular pathways, suggesting that brain specific properties could arise through pathways that have yet to be characterized. Interestingly, SMC proteins did not appear for significant numbers of interaction networks. We speculate that this could be due to a distinct property of SMC or that SMC protein interaction networks have been less extensively studied than those of EC.

In conclusion, we present the curation of data from the Human Protein Atlas that specifically identifies vascular proteins in the human brain. This study identifies both EC and SMC proteins of interest that may be useful for understanding the unique physiology and pathology of the cerebrovascular system. In this initial analysis, we demonstrate broad inter- and intra-organ heterogeneity of brain EC markers and the relatively conserved protein content between brain and non-brain VSMC.

## Supporting information

S1 Fig Examples of EC-specific staining in human brain. We show three examples of validation of EC staining in human brain (from patients with small vessel disease, CADASIL). Stains are shown for (A, B) CLPS (Colipase, pancreatic), (C and D) MYADML2 (Myeloid-associated differentiation marker-like 2), and (E and F) PRX(Periaxin). These markers showed capillary EC staining (see A, C and E) and EC staining in small vessels, the signal was not detected in SMC (B, D and F). Normal brain and images from the Human Protein Atlas showed similar staining distribution. Scale bar represents 100 um.
(PDF)

S2 Fig Examples of EC-SMC staining in human brain. We show three examples of validation of EC-SMC staining in human brain (from patients with small vessel disease, CADASIL). Stains are shown for (A, B) EVI5L(Ecotropic viral intergration site 5-like), (C and D) GTSE1(G-2 and S-phase expressed 1) and (E and F) UBTD1(Ubiquitin domain containing 1). These markers showed capillary EC staining and SMC staining (see small arteries in A, C, E, and F) that was alsoseen in normal brain and images from the Human Protein Atlas. Scale bar represents 100 um.
(PDF)

S3 Fig Examples of SMC staining in human brain. We show three examples of validation of SMC staining in human brain (from patients with small vessel disease, CADASIL). Stains are shown for (A, B) C10orf116(ADRIF, Adipogenesis regulatory factor), (C and D) GPX8 (Glutathione peroxidase 8), and (E and F) RHOF(Ras homolog family member F in filopodia). These markers showed SMC staining (see small arteries in A, C, and E) amd absence of staining in EC of small arteries and capillaries (B, D, and F). This pattern matched that in normal brain and images from the Human Protein Atlas. Scale bar represents 100 um.
(PDF)

S1 File Scoring results from each of the proteins examined in the protein atlas. This file lists the number of antibodies that were used, the number of cortex samples reviewed per protein, and the number of samples for which astrocyte, vessel or neuronal staining was found. A binary score is also shown (0 for not present and 1 for present).
(XLSX)
