# Supplementary material for: Large-scale identification of human cerebrovascular proteins: Inter-tissue and intracerebral vascular protein diversity
Source: PLoS One. 2017 Nov 30;12(11):e0188540. doi: 10.1371/journal.pone.0188540 (PMC5708641; doi:10.1371/journal.pone.0188540)
Supplement: S1 Fig — We show three examples of validation of EC staining in human brain (from patients with small vessel disease, CADASIL). Stains are shown for (A, B) CLPS (Colipase, pancreatic), (C and D) MYADML2 (Myeloid-associated differentiation marker-like 2), and (E and F) PRX(Periaxin). These markers showed capillary EC staining (see A, C and E) and EC staining in small vessels, the signal was not detected in SMC (B, D and F). Normal brain and images from the Human Protein Atlas showed similar staining distribution. Scale bar represents 100 um. (PDF) [file pone.0188540.s001.pdf]

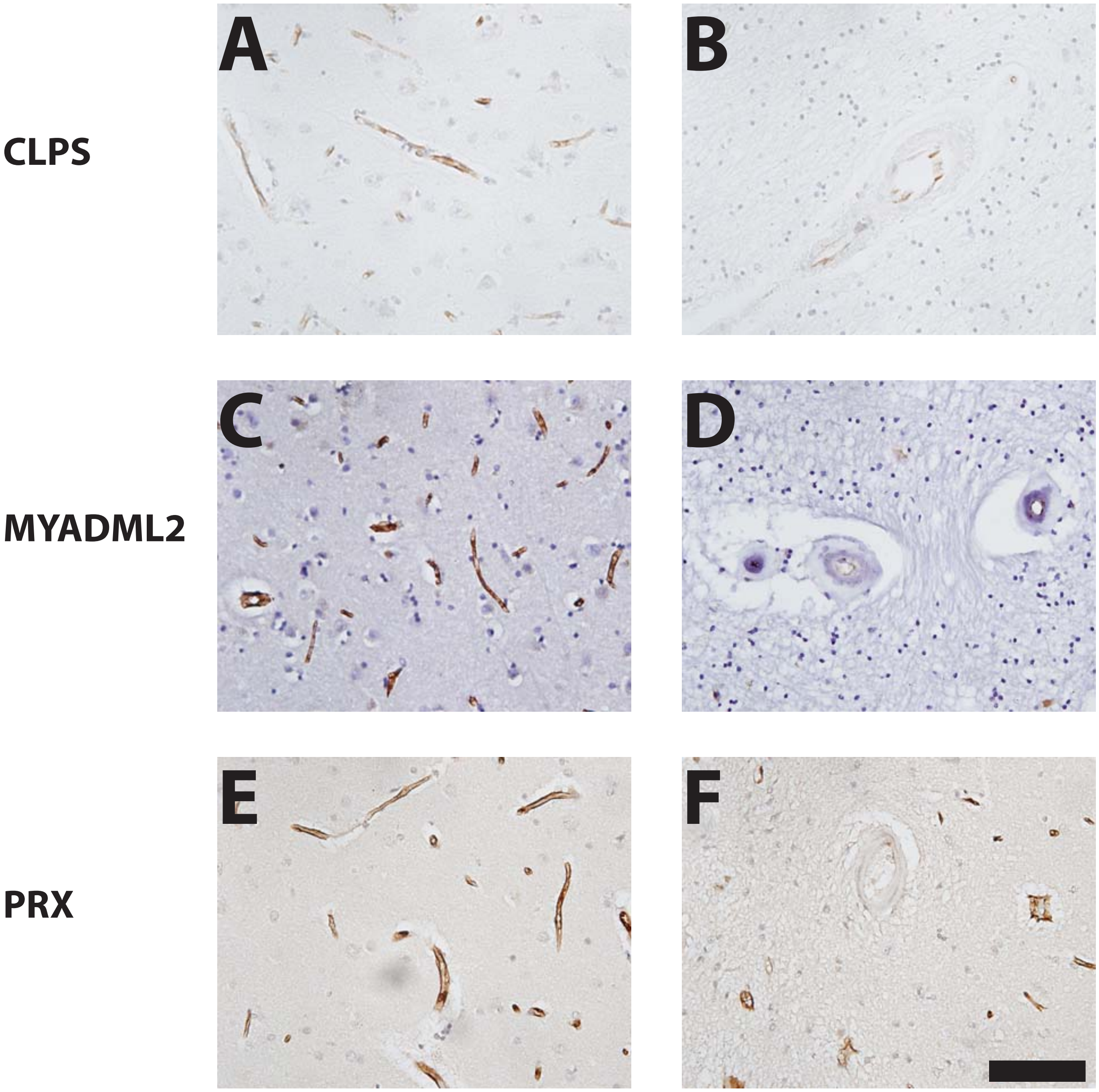

S1 Fig. Examples of EC-specific staining in human brain.
We show three examples of validation of EC staining in human brain (from patients with small vessel disease, CADASIL). Stains are shown for (A, B) CLPS (Colipase, pancreatic), (C and D) MYADML2 (Myeloid-associated differentiation marker-like 2), and (E and F) PRX(Periaxin). These markers showed capillary EC staining (see A, C and E) and EC staining in small vessels, the signal was not detected in SMC (B, D and F). Normal brain and images from the Human Protein Atlas showed similar staining distribution. Scale bar represents 100 um.
