# Supplementary material for: Large-scale identification of human cerebrovascular proteins: Inter-tissue and intracerebral vascular protein diversity
Source: PLoS One. 2017 Nov 30;12(11):e0188540. doi: 10.1371/journal.pone.0188540 (PMC5708641; doi:10.1371/journal.pone.0188540)
Supplement: S2 Fig — We show three examples of validation of EC-SMC staining in human brain (from patients with small vessel disease, CADASIL). Stains are shown for (A, B) EVI5L(Ecotropic viral intergration site 5-like), (C and D) GTSE1(G-2 and S-phase expressed 1) and (E and F) UBTD1(Ubiquitin domain containing 1). These markers showed capillary EC staining and SMC staining (see small arteries in A, C, E, and F) that was alsoseen in normal brain and images from the Human Protein Atlas. Scale bar represents 100 um. (PDF) [file pone.0188540.s002.pdf]

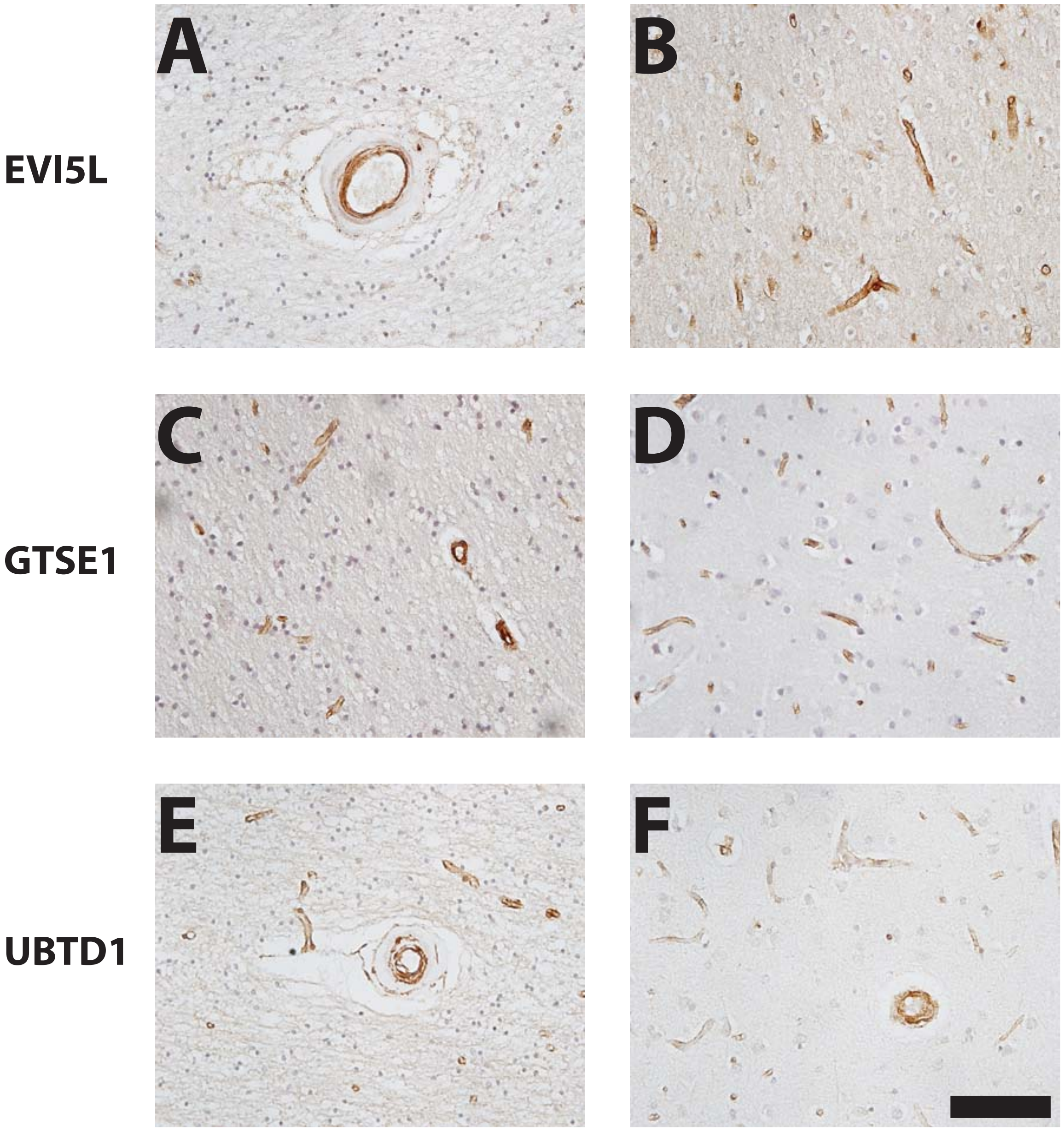

S2 Fig. Examples of EC-SMC staining in human brain.
We show three examples of validation of EC-SMC staining in human brain (from patients with small vessel disease, CADASIL). Stains are shown for (A, B) EVI5L(Ecotropic viral intergration site 5-like), (C and D) GTSE1(G-2 and S-phase expressed 1) and (E and F) UBTD1(Ubiquitin domain containing 1). These markers showed capillary EC staining and SMC staining (see small arteries in A, C, E, and F) that was alsoseen in normal brain and images from the Human Protein Atlas. Scale bar represents 100 um.
