# Supplementary material for: Large-scale identification of human cerebrovascular proteins: Inter-tissue and intracerebral vascular protein diversity
Source: PLoS One. 2017 Nov 30;12(11):e0188540. doi: 10.1371/journal.pone.0188540 (PMC5708641; doi:10.1371/journal.pone.0188540)
Supplement: S3 Fig — We show three examples of validation of SMC staining in human brain (from patients with small vessel disease, CADASIL). Stains are shown for (A, B) C10orf116(ADRIF, Adipogenesis regulatory factor), (C and D) GPX8 (Glutathione peroxidase 8), and (E and F) RHOF(Ras homolog family member F in filopodia). These markers showed SMC staining (see small arteries in A, C, and E) amd absence of staining in EC of small arteries and capillaries (B, D, and F). This pattern matched that in normal brain and images from the Human Protein Atlas. Scale bar represents 100 um. (PDF) [file pone.0188540.s003.pdf]

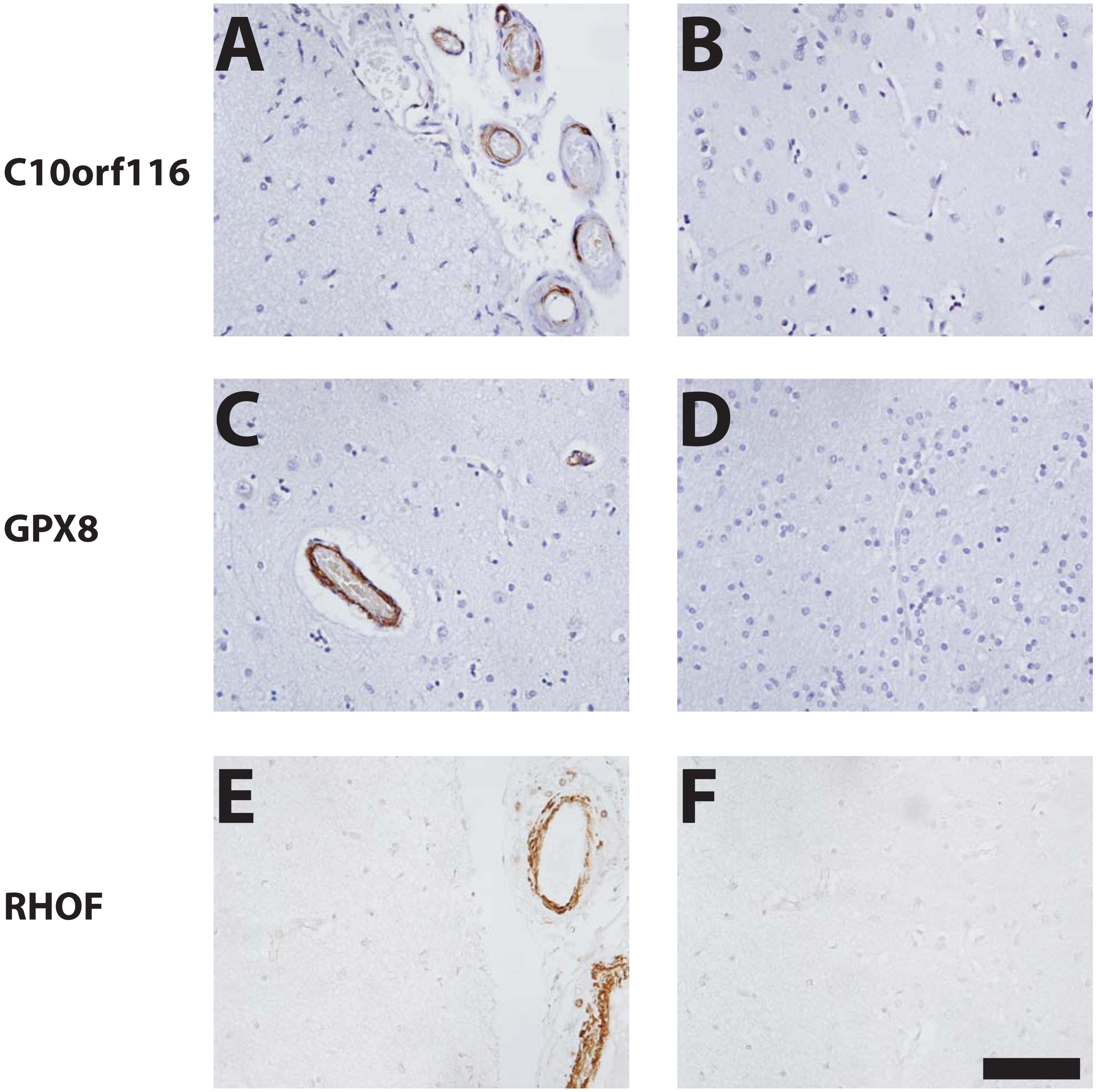


**S3 Fig.** Examples of SMC staining in human brain.
We show three examples of validation of SMC staining in human brain (from patients with small vessel disease, CADASIL). Stains are shown for (A, B) C10orf116(ADRIF, Adipogenesis regulatory factor), (C and D) GPX8 (Glutathione peroxidase 8), and (E and F) RHOF(Ras homolog family member F in filopodia). These markers showed SMC staining (see small arteries in A, C, and E) amd absence of staining in EC of small arteries and capillaries (B, D, and F). This pattern matched that in normal brain and images from the Human Protein Atlas. Scale bar represents 100 um.
